# Discovery of estrogen receptor α target genes and response elements in breast tumor cells

**DOI:** 10.1186/gb-2004-5-9-r66

**Published:** 2004-08-12

**Authors:** Chin-Yo Lin, Anders Ström, Vinsensius Berlian Vega, Say Li Kong, Ai Li Yeo, Jane S Thomsen, Wan Ching Chan, Balraj Doray, Dhinoth K Bangarusamy, Adaikalavan Ramasamy, Liza A Vergara, Suisheng Tang, Allen Chong, Vladimir B Bajic, Lance D Miller, Jan-Åke Gustafsson, Edison T Liu

**Affiliations:** 1 Genome Institute of Singapore, Singapore 117528; 2 Center for Biotechnology, Karolinska Institute, Novum, S-141 57 Huddinge, Sweden; 3 Knowledge Extraction Lab, Institute for Infocomm Research, Singapore 119613; 4 Department of Medical Nutrition, Karolinska Institute, Novum, S-141 86 Huddinge, Sweden

## Abstract

**Background:**

Estrogens and their receptors are important in human development, physiology and disease. In this study, we utilized an integrated genome-wide molecular and computational approach to characterize the interaction between the activated estrogen receptor (ER) and the regulatory elements of candidate target genes.

**Results:**

Of around 19,000 genes surveyed in this study, we observed 137 ER-regulated genes in T-47D cells, of which only 89 were direct target genes. Meta-analysis of heterogeneous *in vitro *and *in vivo *datasets showed that the expression profiles in T-47D and MCF-7 cells are remarkably similar and overlap with genes differentially expressed between ER-positive and ER-negative tumors. Computational analysis revealed a significant enrichment of putative estrogen response elements (EREs) in the *cis*-regulatory regions of direct target genes. Chromatin immunoprecipitation confirmed ligand-dependent ER binding at the computationally predicted EREs in our highest ranked ER direct target genes, *NRIP1*, *GREB1 *and *ABCA3*. Wider examination of the *cis*-regulatory regions flanking the transcriptional start sites showed species conservation in mouse-human comparisons in only 6% of predicted EREs.

**Conclusions:**

Only a small core set of human genes, validated across experimental systems and closely associated with ER status in breast tumors, appear to be sufficient to induce ER effects in breast cancer cells. That *cis*-regulatory regions of these core ER target genes are poorly conserved suggests that different evolutionary mechanisms are operative at transcriptional control elements than at coding regions. These results predict that certain biological effects of estrogen signaling will differ between mouse and human to a larger extent than previously thought.

## Abstract 2

Microarray analysis has identified 89 estrogen target genes. The *cis*-regulatory elements found upstream of those genes are not well conserved in mouse and human.

## Background

Estrogens are involved in a number of vertebrate developmental and physiological processes. Human and animal studies have revealed the roles of estrogen receptor (ER) in female and male sexual development and behavior, reproductive functions, and the regulation of the neuroendocrine and cardiovascular systems and bone metabolism [1]. Molecular characterizations of breast tumors and epidemiological studies have also shown important roles for estrogens and ERs in the genesis, progression, and treatment of breast cancers [2,3].

Two ER subtypes, ERα and ERβ, are known to mediate estrogen signaling; and they function as ligand-dependent transcription factors [4]. After traversing the cellular membrane, estrogens bind to the receptors, leading to receptor activation. ERs interact with *cis*-regulatory elements of target genes either directly by binding to previously described conserved estrogen response elements (EREs; 5'-GGTCANNNTGACC-3', where N is any nucleotide) or indirectly by associating with AP-1 and Sp1 transcription factor complexes and their respective binding sites [5-9]. Co-activators and co-repressors form complexes with ERs and are involved in regulating estrogen responses [10]. The cyclical turnover of ER and transcriptional complexes at the regulatory elements of target genes also presents an additional regulatory mechanism [11-13]. Tissue-specific distribution of co-regulators, associated transcription factor complexes, and receptor subtypes and splice variants are potential mechanisms for the observed pleiotropic effects of estrogens [14]. At the molecular level, the consequence of ER activation appears to be alterations in transcriptional activity and expression profiles of target genes. A number of genes, including those for trefoil factor 1/pS2, cathepsin D, cyclin D1, c-Myc and progesterone receptor, are positively regulated by ERα [15-20]. Transcriptional repression by ERs has been documented but is not as well studied or understood.

Microarray experiments have been carried out, particularly in breast tumor cell lines, to study alterations in gene-expression profiles in response to estrogen treatment [21-27]. Many key issues remain to be addressed, however, using these initial inventories of responsive genes, including overall conservation of responses across cell lines, *in vivo *relevance in breast tumors, and *cis*-regulatory element mapping and molecular characterization and confirmation of the interaction between ER and putative target genes. In this study, we took a combinatorial approach to ERα target gene discovery and characterization by using high-density DNA microarrays to obtain a global gene-expression profile of hormone response in ERα-positive (EPα^+^) breast tumor cells. This included drug treatments that interrogate ER-mediated and translation-independent regulation, integration of additional *in vitro *estrogen-response data and human breast tumor sample gene-expression data for candidate gene validation and identification of relevant *in vivo *targets, computational binding site modeling and promoter analysis to map putative ER-binding sites, and chromatin immunoprecipitation (ChIP) to characterize the interaction between ER and the regulatory elements of candidate target genes. Here we present our findings and discuss the insights they provide into the genome-wide architecture of the ER-mediated transcriptional regulatory network and its conservation in cell lines, breast tumors and through evolution.

## Results

### Global gene expression profile of estrogen response

**Figure 1:**
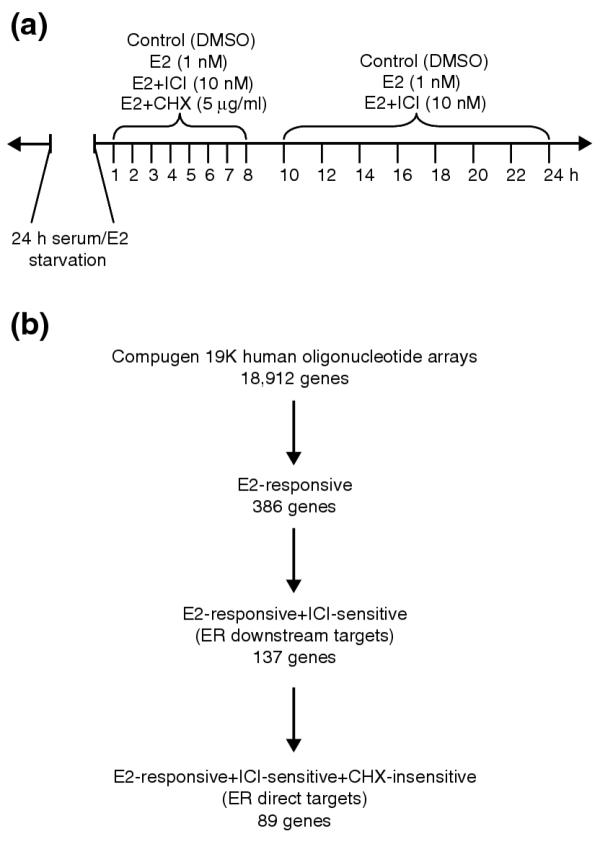
Experimental design and microarray data selection of the time-course analysis of estrogen response in T-47D cells. **(a) **Cells were starved of serum and estrogen for 24 h before treatment with dimethysulfoxide (DMSO; carrier control), 17β-estradiol (E2), and E2 in combination with ICI 182,780 (ICI) and cycloheximide (CHX). Samples were taken at the 16 time points indicated. **(b) **Procedure for identifying direct ER targets. Data selection for estrogen-responsive genes was based on *p*-value cutoffs (*p *≤ 0.052) and magnitude of response (at least 1.2-fold change in the same direction for three time points). ICI sensitivity and CHX insensitivity were also determined by statistical measures, *p *≤ 0.058 and *p *≥ 0.24 respectively. Cutoff values were informed by expression profiles of cathespsin D and progesterone receptor, both known to be regulated by ER.

High-density DNA microarrays are powerful tools that simultaneously determine the transcriptional profiles of thousands of genes and are especially well suited for studies of transcription factor function. Previous efforts to determine changes in gene expression profiles following hormone treatment in MCF-7 [21-25,27] and ZR-75-1 [26] ER^+ ^breast carcinoma cell lines have yielded a number of novel estrogen-responsive genes and demonstrated the utility of such genome-scale technologies in studying estrogen biology. These earlier studies, however, only included anti-estrogen and cycloheximide (CHX) treatments either at a limited number of time points or only in validation assays for a handful of putative responsive genes. Therefore, to map more comprehensively the transcriptional regulatory network regulated by ER and to generate data in an additional ER^+ ^breast tumor cell context for cross-cell line analysis, we treated the estrogen-dependent T-47D ER^+ ^breast cancer cell line with 17β-estradiol (E2) and with E2 in combination with either the pure anti-estrogen ICI 182,780 (ICI) or the protein synthesis inhibitor CHX and performed high-resolution time-course gene-expression analyses (see Figure 1a for treatments and time points) using spotted oligonucleotide (60-mers) microarrays containing probes representing around 19,000 human genes. The concentrations of E2 (1 nM) and ICI (10 nm) used in this study were sufficient to respectively drive and inhibit hormone-responsive cell proliferation. T-47D cells differ in karyotypic abnormalities and nuclear receptor co-regulator expression levels from cell lines previously used - MCF-7 and ZR-75-1 - but have the advantage of expressing ER at more physiologic levels [28,29]. Samples were harvested on an hourly basis for the first 8 hours (0-8 hours) following hormone treatment and bi-hourly for the next 16 hours (10-24 hours) for a total of 16 time points surveyed (Figure 1a).

Estrogen-responsive genes were determined by statistical analysis of expression ratios in E2-treated samples versus the mock-treated controls using a two-tailed paired *t*-test with *p*-value cutoff. In addition, the genes were filtered for at least a 1.2-fold change in the same direction in three or more time points. Our choice of data-selection criteria was informed by the observed expression levels and profiles of known hormone-responsive and ER target genes such as the progesterone receptor, cathepsin D and stanniocalcin 2. Responsive genes were selected for E2 responsiveness (*p *< 0.052) and filtered for ICI sensitivity (*p *< 0.057) and CHX insensitivity (*p *> 0.24) by comparing E2-treated samples with E2+ICI and E2+CHX samples to isolate putative ER downstream targets and direct targets, respectively. Figure 1b summarizes the statistics of the selection process. Expression profiles of estrogen-responsive genes were visualized by Eisen clustergrams and the genes were sorted by hierarchical clustering [30].

**Figure 2:**
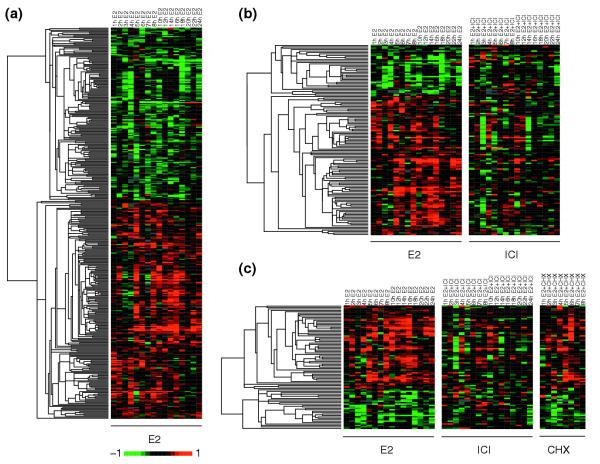
Expression profiles of estrogen-responsive genes. **(a) **The 386 genes responsive to E2 were visualized by hierarchical clustering and the Eisen TreeView software. The columns represent time points arranged in chronological order, and each row represents the expression profile of a particular gene. By convention, upregulation is indicated by a red signal and downregulation by green. The magnitude of change is proportional to the brightness of the signal. Each panel represents a treatment condition as noted in the column headings. **(b) **ICI and **(c) **CHX treatments were included to identify the 137 ER-regulated genes and 89 primary response genes, respectively.

For estrogen-responsive genes (Figure 2a), the expression profiles clustered into two groups: genes that are upregulated by hormone treatment (Figure 2a, red) and those that are downregulated (Figure 2a, green). Of the responsive genes, 58.5% (226/386) were upregulated following estrogen treatment. Figure 2b shows the expression profiles of the 137 genes specifically regulated by ER (defined by being responsive to estrogen and blocked by ICI treatment, Figure 2b, right panel). These genes cluster in a similar fashion as the estrogen-responsive genes. A notable finding is that ER-regulated genes, as determined by E2 response and ICI sensitivity, account for only 35.5% (137/386) of all estrogen-responsive genes, suggesting the possibility of ER-independent signal transduction mechanisms in mediating transcriptional responses to hormone exposure. Interestingly, ICI appears to have a greater effect on E2-upregulated than downregulated genes as these upregulated genes account for 71.5% (98/137) of the ICI-sensitive subset. Eighty-nine primary response genes constituted the putative ER direct targets - defined as responsive to hormone treatment, sensitive to ICI but not affected by CHX treatment (Figure 2c, right panel). From these observations the number of direct target genes involved in initiating hormone response in breast tumor cells might represent only 0.47% (89/18,912) of the genes in the human genome. The list of putative ER target genes is presented in Table 1, and the complete listing of E2-responsive genes is given in Additional data file 4. Genes previously shown to be ER targets are shown in bold type (see Table 1). We note that five of the direct target genes on our list correspond to the results presented in the only other published microarray study to include anti-estrogen and CHX treatments as part of the experimental design [26]. The discrepancy between the two datasets is likely to be due to the differences in cell lines, experimental designs, array platform and the filtering parameters used for selecting target genes. For example, the Soulez and Parker study [26] utilized ZR-75-1 cells tested at only two time points (6 and 24 hours). Moreover, the Affymetrix HuGeneFL arrays used in that study contained probes for 5,600 genes as compared to the nearly 19,000 genes on our arrays, and represented early array technology with known limitations. Similarly, the absence of known target genes TFF1/pS2 and cyclin D1 in our list of direct targets is probably due to differences in the transcriptional cofactors and genomic abnormalities in the T-47D cells. The absence of the c-*MYC *proto-oncogene on our list is probably because the probe was not represented in the arrays used in our study.

To control for the confounding direct effects of ICI and CHX, independent of E2, we also treated the cells with only ICI for 2, 8, 12 or 24 hours, or only CHX for 2 or 8 hours. CHX treatment partially obscured the responses in 4 of 89 genes (4.5%) that met the selection criteria for putative direct target genes by inducing a detectable E2-like effect following either CHX, E2, or E2+CHX treatments (see Additional data files 5 and 6). As this result does not rule out these genes as direct targets, we included them in the direct target list, but noted this caveat in Table 1. CHX treatment alone had the opposite effect to E2 treatment in 32 (8.3%; 32/386) of the E2-responsive genes. However, in the presence of the hormone and the drug together (E2+CHX), CHX did not antagonize the E2 response of these genes and therefore did not affect the selection of putative direct targets. ICI treatment alone elicited an unexpected E2-like response (that is, identical response following either E2, ICI or E2+ICI treatments) in nine (2.3%; 9/386) E2 responsive genes. Because these nine genes did not meet the selection criteria for ICI antagonism, the putative direct target genes were also not affected. Thus, the independent effects of ICI and CHX did not substantially alter the final gene list or conclusions in our analysis.

### Comparison of T-47D and MCF-7 estrogen-response profiles

A number of breast cancer cell lines have been used in *in vitro *studies of estrogen responses, but most of the data, including those from published microarray studies, have come from experiments using MCF-7 cells. Therefore, we were interested in comparing the expression profiles in response to hormone treatment between MCF-7 cells and the T-47D cells used in our studies. For our analysis, we obtained a publicly available MCF-7 hormone and tamoxifen response dataset [31] from the Stanford Microarray Database [32].

**Figure 3:**
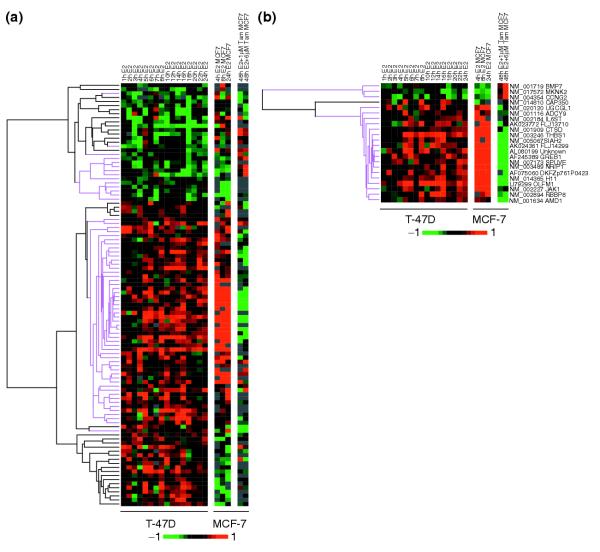
Comparative analysis of estrogen-responsive gene expression profiles in T-47D and MCF-7 breast cancer cell lines revealed similar responses. **(a) **Expression data of 103 genes that were responsive to E2 and sensitive to ICI treatment in T47-D cells and present in the MCF-7 dataset [31] revealed concordant responses to E2 in 64% (66/103) of the genes (highlighted in magenta). **(b) **If the MCF-7 data is selected for E2-responsive and tamoxifen (Tam)-sensitive genes (see Materials and methods), there is a 24-gene overlap between the two datasets and the responses to E2 and the anti-estrogens ICI or tamoxifen are highly concordant (magenta) at nearly 96% (23/24).

Using Unigene cluster IDs from build 166 as the common identifiers between the two datasets, we extracted expression data from 104 of 137 T-47D ER-regulated genes (Figure 3a) that were also present in the MCF-7 dataset. For genes with multiple entries in the MCF-7 data, the entry with either the most complete data or with similar expression profiles to the T-47D results was selected for analysis. Overall, the results from the MCF-7 experiments correspond to the majority (64%; 66/103) of expression profiles of responsive genes obtained in the T-47D cells as defined by same direction changes in the available data points in MCF-7 cells following hormone treatment (see Figure 3a). Using stringent selection criteria for the MCF-7 data for E2 response and sensitivity to tamoxifen treatment (see Materials and methods), we found 24 genes that can be defined as ER regulated in both MCF-7 and T-47D datasets. Remarkably, there was high concordance between the two studies with 23 out of 24 (96%) of the genes showing concordance in their expression response to estrogen (Figure 3b). In contrast, there was little concordance in microarray datasets from unrelated stem-cell studies despite use of similar experimental systems and identical array platforms [33]. These findings were further validated by the many overlaps between the genes identified in this investigation and the estrogen-responsive genes reported by Frasor and colleagues (data not shown) [23]. The similarities we observe between the two ER studies with different experimental designs and array platforms suggest that the two ER^+ ^cell lines share common estrogen-response pathways.

### Differential expression of putative ER-regulated genes in breast tumors

A key question we wished to address was whether the *in vitro *observations in cell lines reflected biological significance *in vivo*. To address this, we explored the association between the ER-regulated genes identified in our *in vitro *analysis and the ER status-associated expression profiles in breast tumor samples. We hypothesized that putative ER target genes should be differentially expressed in breast tumors in an ER status-dependent manner. For example, pS2/TFF1 and cyclin D1, both upregulated by estrogen treatment in MCF-7 cells, were shown to be expressed at higher levels in ER^+ ^tumors [34,35].

**Figure 4:**
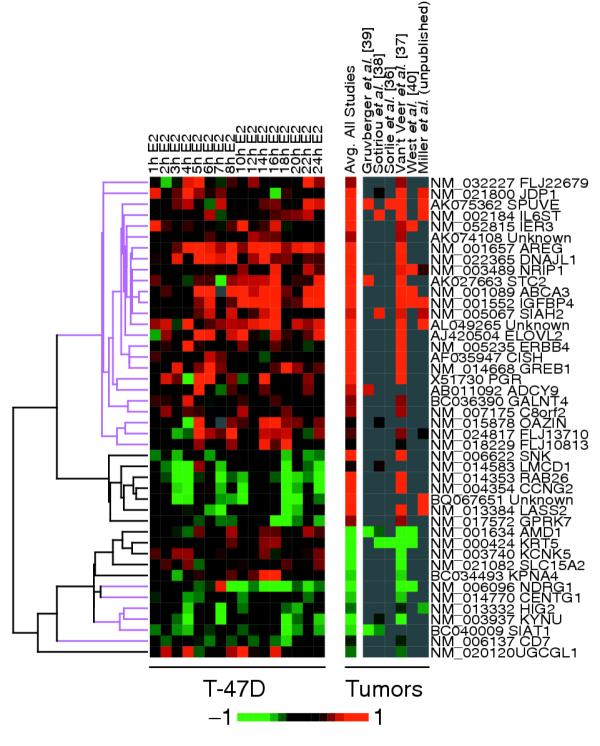
Estrogen-responsive genes identified in cell-line studies were also differentially expressed in ER^+ ^breast tumors compared to ER^- ^tumors. Estrogen-responsive gene-expression profiles are compared to the composite expression ratio for those genes in each of the six breast tumor studies surveyed (L.D.M., B.M.F. Mow, L.A.V., and E.T.L., unpublished work and [36-40]). Differentially expressed genes were defined by *p *< 0.01 between ER^+ ^and ER^- ^tumor samples. Genes that responded similarly in ER^+ ^tumors and *in vitro *following E2 treatment are highlighted in magenta.

A number of breast cancer microarray studies have shown that ER status remains the most important prognostic marker and tumor classifier. Expression data from six breast cancer microarray studies (L.D.M., B.M.F. Mow, L.A.V., and E.T.L., unpublished work and [36-40]) were mined for genes that were differentially expressed (*p*-value < 0.01 false discovery rate, ER^+ ^vs ER^-^) in human breast tumor samples with respect to ER status. Of the 137 ER-regulated genes (E2 responsive, ICI sensitive) identified in the T-47D study, 44 genes were differentially expressed in at least one breast cancer study (Figure 4). The 44 ER-regulated genes represent only about 1% (44/3811) of the 3,812 ER-status-associated genes that met the selection criteria (*p *< 0.01 in one or more studies), suggesting that the estrogen-responsive pathways represent only a minor part of the ER-status-associated transcriptome in breast tumors. This is similar to observations made previously by Meltzer and co-workers [22,39]. However, there appears to be a significant enrichment of ER-regulated genes within the ER-status-associated genes compared to the frequency of these ER-regulated genes represented in the microarray used in our study (1.15%, 44/3,811 vs 0.72%, 137/18,912, *p *= 0.006 by chi-square analysis).

To compare the expression profiles of responsive and differentially expressed genes, we plotted the average relative expression ratios of each gene (ER^+^/ER^-^) across all samples from the breast cancer studies (Figure 4). There was surprising concordance (70.5%; 31/44) between the estrogen-responsive genes identified in T-47D cells and genes differentially expressed in breast tumors. For example, genes upregulated by hormone treatment (Figure 4, left panel, red) were also overexpressed in ER^+ ^breast tumors (Figure 4, right panel, red). We noted a subset (29.5%; 13/44) of genes that exhibited opposite responses following estrogen treatment *in vitro *as compared to the ER-status-associated expression in tumors. These 13 genes that are discordant between cell line and tumor data were, however, consistent across the two cell lines (T47-D and MCF-7). This suggests context-dependent regulation of some downstream pathways, which is likely to be different between primary tumors and experimental cell lines. Taken together, we note that these *in vitro *validated estrogen-responsive genes are also differentially expressed in ER^+ ^primary tumors, and may therefore have direct biological and clinical significance.

### Computational modeling and predictions of ER-binding sites

Previous studies have identified the consensus ERE and the AP-1- or Sp1-binding sites in DNA as possible target motifs for. This would suggest that the 89 direct responding genes should be enriched for these binding motifs within the transcriptional control regions. To further explore this, we computationally extracted sequences flanking (-3,000 to +500) the transcriptional start site (TSS, see Materials and methods section), defined as the most 5' nucleotide of the reference transcript in the NCBI RefSeq database, of candidate genes and queried them for potential ER-binding sites. The size and locations of the sequences flanking the start sites were selected because most of the characterized ER-binding sites have been mapped to these regions in known target genes [5].

**Figure 5:**
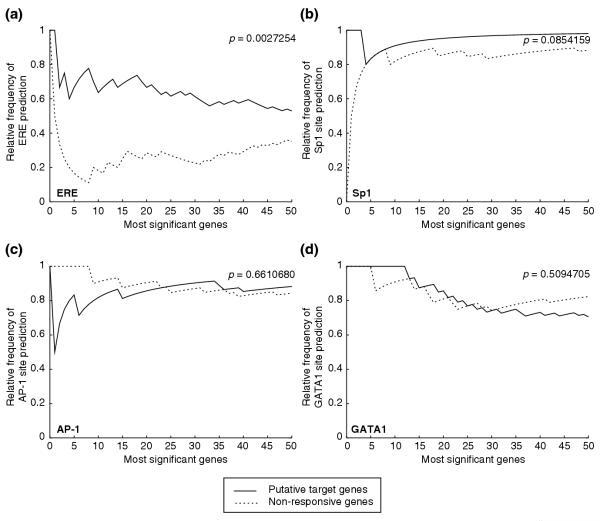
ERE-like sequences are enriched in the extended promoter regions of putative target gene. **(a) **ERE predictions were made using a previously published model and optimized sensitivity setting. Prediction models were tested on the extended promoter regions (-3,000 to +500, both strands) from the most significant putative ER target and non-responsive genes, ordered by statistical significance. The *y*-axis represents the relative frequency of binding-site predictions as determined by the number of genes with predicted sites divided by the total number of genes. The number of most significant genes queried is indicated on the *x*-axis. Frequency of ERE predictions in putative target genes is significantly greater (*p *= 0.0027) than the similarly ranked non-responsive genes. Binding-site predictions were also carried out using position weight matrices describing sites for **(b) **Sp1, **(c) **AP-1 and **(d) **GATA1. Both Sp1 and AP-1 are known to be involved in regulating ER binding in certain target genes. GATA1 sites were included as negative controls. There is no significant enrichment of these sites in the putative target genes (see *p*-values in figure).

For binding-site predictions we used our previously described ERE model [41] and AP-1 and Sp1 binding-site position weight matrices from the TRANSFAC database [42]. We also included the binding site for the GATA1 transcription factor as a negative control as it is not known to be involved in ER binding. Model sensitivities for all the sites surveyed were set at the established optimal setting for the ERE model of 83% sensitivity in detecting known binding sites in the training data for the models. Figure 5 shows the performance of the ERE (Figure 5a), AP-1 (Figure 5b), Sp1 (Figure 5c), and GATA1 (Figure 5d) binding-site models. The *y*-axis for each graph represents the relative frequency of binding-site prediction as determined by the fraction of genes with predicted binding sites over the total number of genes queried; the *x*-axis represents the number of most significant genes investigated, ordered by statistical significance, for each of the groups of genes (see Materials and methods). Since short binding site motifs are ubiquitous in the human genome, we asked whether there was enrichment of such response elements in the 3.5 kilobase (kb) upstream windows of responsive genes as compared to unresponsive genes. Enrichment for each motif is represented by a divergence of the relative frequencies of binding-site predictions for putative target genes (Figure 3, solid lines) and non-responsive genes (Figure 3, fragmented lines). For ERE predictions, we observed a threefold enrichment of putative sites in the 10 most significant primary response genes as compared to the most non-responsive controls (Figure 3a), and twofold and approximately 70% enrichment for the 25 and 50 most significant genes, respectively. Overall, the enrichment of ERE sites in putative ER direct target genes is statistically significant (*p *= 0.0027). The enrichment of putative Sp1 sites in the target genes was more modest but did not reach statistical significance (12.5% enrichment for the 10 most significant target genes; *p *= 0.085). This is expected as Sp1 sites are quite common in the human genome and additionally function in general transcriptional regulation. We did not observe any enrichment of AP-1 sites (*p *= 0.66) or the negative control GATA1 sites (*p *= 0.51). These findings suggest that the ERE is the major response element mediating the specific regulation of ER target genes on a whole-genome scale. We also surmised that although Sp1 and AP-1 binding sites are known to facilitate ER functions in some target genes they are not used as a common ER-targeted *cis*-regulatory element within the human genome, at least not sufficiently to distinguish target genes from non-responsive genes.

To determine the conservation and potential functionality of the predicted EREs, we also examined the same 3.5 kb window in the 5' upstream regions of mouse orthologs of the 89 putative human ER target genes. Seventy-two human-mouse orthologous gene pairs were extracted from the Mouse Genome Database [43] and the regulatory regions demarcated and analyzed for potential EREs as described for the human sequences (see Materials and methods). We then compared the ERE predictions from the two organisms for the following features: conservation of the core ERE half-sites (GGTCANNNTGACC), excluding the flanking purine bases, between the two most similar sequences when multiple EREs are predicted in either organism; conservation of the 20 bases flanking the 5' and 3' ends (40 bases total) of the predicted EREs; and the distance between the binding-site sequences and the TSS.

**Figure 6:**
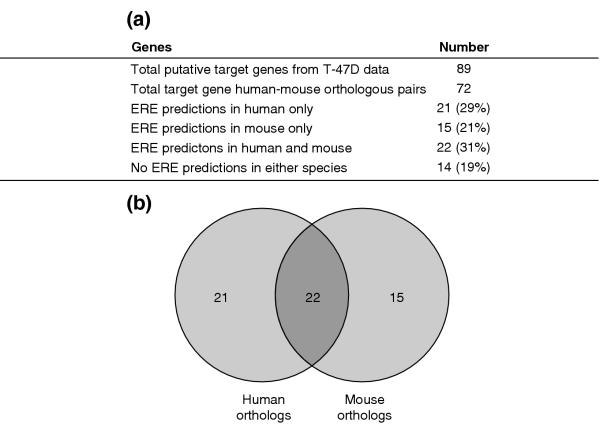
Comparison of human and mouse orthologs. **(a) **Statistics from the comparative analysis of predicted EREs in human and mouse orthologous putative target gene pairs. **(b) **Venn diagram showing that out of the 72 orthologous pairs extracted for analysis, only 22 pairs have ERE predictions made in both the human and mouse sequences.

The statistics of our analysis is summarized in Figure 6a. Of the orthologous mouse-human pairs, 81% (58/72) have at least one ERE prediction and 22 (31%; 22/72) gene pairs have ERE predictions in both organisms. However, of the human direct target genes, 29% (21/72) have no EREs upstream of the mouse orthologs. Conversely, 21% (15/72) of the mouse genes with EREs have no ERE upstream of their human orthologs. Of the 22 gene pairs that have ERE predictions in both organisms (see Venn diagram in Figure 6b), only four have perfect conservation of the core ERE sequences (Table 2). These four perfectly conserved ERE pairs also have the highest conservation in their flanking sequences (average identity = 74%) and the smallest difference in the relative positions of binding sites (average difference Δ*d *= 469 bases) between the human and the mouse sequences. In fact, the relative positions of the conserved EREs only differ by an average of 52 bases if the predicted EREs for *GREB1 *(human, NM_014668; mouse, NM_015764), which differed by 1.7 kb in their relative position, were excluded from the analysis. For the ERE mouse-human pairs with one or more base deviations in their core sequences, there is little conservation in the flanking sequences and in the relative positions of predicted EREs (see Table 2). These findings indicate that although the ERE motif is conserved through evolution, specific EREs found in the 5' regulatory regions of target genes are rarely conserved. They also suggest potential differences in the molecular mechanisms of ER function and in the repertoire of target genes between human and rodents. In light of this, our inference of ER function in humans from the results obtained from animal studies may warrant a re-evaluation and additional validation.

### Validation of direct ER target genes by chromatin immunoprecipitation

**Figure 7:**
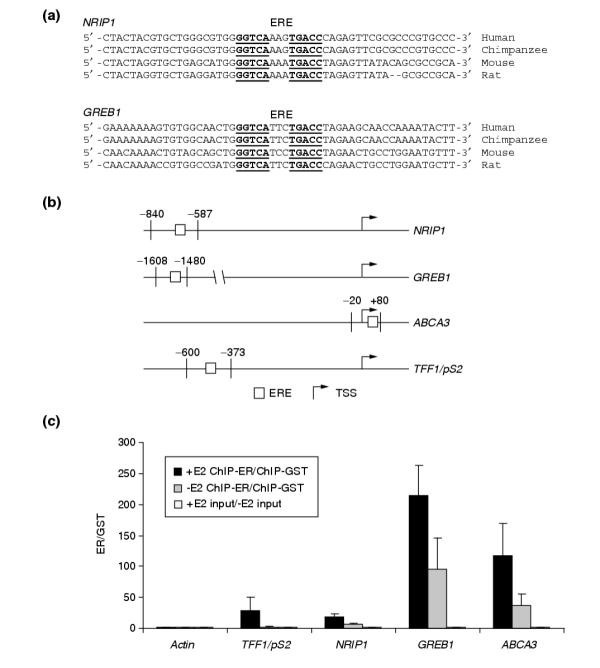
ER binds promoter regions encoding both conserved and non-conserved predicted response elements in an estrogen-dependent manner. **(a) **EREs (underlined) found upstream of *NRIP1 *and *GREB1 *coding regions are conserved in human, chimpanzee, mouse, and rat genomes. **(b) **PCR primers flanking the predicted conserved (*NRIP1 *and *GREB1*) and non-conserved (*ABCA3 *and *TFF1/pS2*) EREs were designed to detect ER binding following ChIP assays. The relative positions of the primers and ERE, relative to the TSS, are indicated. **(c) **Interactions between ER and predicted EREs were enhanced by estrogen treatment. MCF-7 cells were either mock-treated with the carrier dimethyl sulfoxide (-E2, gray bars) or treated with estradiol (+E2, black bars), followed by ChIP experiments. Black and gray bars indicate the enrichment of the binding site in anti-ER ChIP experiments over anti-GST ChIP experiments. Enrichment of all EREs was observed in hormone-treated cells whereas the mock-treated cells displayed less or very little enrichment. There was no enrichment of actin exon 3 control region or any of the input controls (open bars).

The genomics and informatics approaches have enabled us to identify genes that meet the conventional definition for ER target genes (for example, responsive to E2, sensitive to ICI, and insensitive to CHX), are conserved in ER^+ ^breast cancer cell lines and tumor samples, and encode putative ER-binding sites in the promoter regions. Two genes emerged at the top of the list of direct target genes following these analyses. One was for nuclear receptor-interacting protein 1 (NRIP1), also known as receptor-interacting protein 140 (RIP140), first identified as an ER-binding protein and a co-regulator of receptor activity [44,45]. It was subsequently shown to bind and modulate transcriptional activities of other nuclear receptors [46,47]. Previous microarray experiments in MCF-7 and ZR75-1 cells showed that *NRIP1 *transcript levels were raised following estrogen treatment, and its expression dynamics in the presence of anti-estrogens and CHX were consistent with other primary response genes [23,24,26]. In this study, we have also identified *NRIP1 *as a putative ER target gene that is upregulated by E2, sensitive to ICI treatment and insensitive to CHX in T-47D cells. Furthermore, we detected a conserved perfect ERE at around 700 bases upstream of the TSS, indicating a potential ER-binding site and direct regulation by the activated receptor. The other direct target gene - gene regulated by estrogen in breast cancer 1 (*GREB1*) - was identified in a subtractive hybridization screen for estrogen-responsive genes in MCF-7 cells. It has no known function and does not appear to share significant homology with any other gene in the sequence databases [48]. A perfect ERE was found at around 1.6 kb upstream of the TSS of *GREB1 *and the predicted ERE is also conserved in mouse. Given that both *NRIP1 *and *GREB1 *have been conserved during vertebrate evolution, we compared the 5' upstream regions from human, chimpanzee, mouse and rat genome sequences to see whether the predicted regulatory element has been conserved in additional murine and primate species. For all of the regions surveyed, we found that the core ERE has been perfectly conserved (Figure 7a). In addition, sequences flanking the predicted ERE were also highly conserved, suggesting functionality for these regions.

To determine the role of the predicted ERE as an ER-binding site, we performed chromatin immunoprecipitations (ChIPs) using anti-ER antibodies. In addition to the two conserved EREs, we also included two non-conserved EREs from *TFF1/pS2 *(positive control) and ATP-binding cassette, subfamily A, member 3 (*ABCA3*), a gene related to other ABC transporters that are thought to be involved in cellular lipid transport and which is a putative ER direct target gene as determined in this and a previous study [26]. Forward and reverse primers (Figure 7b) flanking the ERE were designed to specifically detect and quantify genomic DNA fragments that co-precipitate with ER in real-time PCR experiments. Following hormone treatments, we did not observe significant enrichment of the negative control actin exon 3 region in anti-ER precipitates as compared to the anti-GST antibody control or the input genomic DNA from the nuclear lysates for all primer pairs tested (Figure 7c). In contrast, semi-quantitative PCR analysis (see Materials and methods) of the ChIP products using primers flanking the predicted EREs revealed ER binding to these sites in the absence of estrogen and after hormone treatment (see Figure 7c). Furthermore, the binding appeared to be enhanced following estrogen treatment, suggesting a role for activated receptors in mediating the observed transcriptional regulation of these genes. The functionality of the conserved EREs in *NRIP1 *and *GREB1 *was also recently reported in a study of near-consensus EREs in the human and mouse genomes [49].

## Discussion

We have conducted a genome-wide analysis of E2-responsive genes. Through a strategy of iterative validation using genomics, informatics and experimental biology we have identified and characterized a core set of 89 ER direct target genes out of the 18,912 genes represented on our microarray (0.5%). This set of direct target genes derived from experiments in T-47D cells show very similar behavior in another cell line, MCF-7, and also overlap with genes that can distinguish ER status in human breast cancers. Taken together, these results suggest common underlying mechanisms for ER transcriptional control and define specific genetic components of the ER transcriptional network that are consistent across model experimental and clinical conditions.

These results emboldened us to decipher the rules and informational framework underlying ER transcriptional control. The anti-estrogen treatment with the ICI drug and preincubation of the cells with CHX allowed us to identify genes likely to be ER direct targets. We extracted extended promoter regions (- 3,000 bp to +500 bp) and determined potential ER-binding sites by using ERE and AP-1 and Sp1 binding-site models [41,42]. Because transcription factor binding elements occur very frequently in the genome, finding an ERE, AP-1 or Sp1 site only in ER-responsive genes is highly unlikely. Instead, we asked whether the probability of finding an ER-associated response element was significantly higher than that seen in ER-unresponsive genes. Our results, depicted in Figure 5, show distinctly that EREs are enriched in the putative direct estrogen-responsive genes (*p *= 0.0027) but that binding sites for AP1, Sp1 or GATA1 (which we used as a negative control response element) are not. The ERE thus appears to be the predominant ER transcriptional control element. Moreover, despite definitive experiments showing the ability of AP-1 and Sp1 sites to mediate ER responses [7-9,47,50-54], our results suggest that their usage is not a common mechanism for specific ER transcriptional control on a genome-wide scale.

Previous investigations have uncovered a functional ERE embedded within an *Alu *repetitive sequence that is frequent in the genome [55]. Inclusion of this *Alu *ERE into our analysis, however, dramatically degrades the enrichment of EREs found in direct ER-responsive genes (*p *= 0.06). This suggests that though such EREs are experimentally functional, they have little impact on the specific ER transcriptional cassette, functioning as no more than 'noise' in the system. This has been confirmed by negative ChIP data for several *Alu*-ERE sites (data not shown). These observations highlight the potential confounding factors in genome-wide analysis of functionally relevant response elements.

Our use of a 3.5 kb window around the TSS to search for relevant EREs captures the majority of known EREs [5] and represents a liberal survey of 5' regulatory regions. Despite this, we found that only about 50% of the target genes encode ERE-like sequences (including ERE half-sites) in their promoters. It is possible that ER-binding sites outside this window may be involved in regulating the specific activities of ERs. In support of this, Bourdeu and colleagues very recently described the identification and validation of EREs within DNA 10 kb upstream (relative to TSS) and 5 kb downstream in 5' regions of a number of human genes [49], indicating the presence of functional enhancer elements outside the region surveyed in our study. In addition, errors in annotating the TSS or additional 5' exons may account for up to an 8% error rate for TSS determination in known genes and 80% error rate in predicted genes (Y.J. Ruan, E.T.L. and C.L. Wei, unpublished work). Future studies will need to incorporate these information in the ERE analyses.

Given that *in silico *identification of EREs does not assure their function in an ER response, we selected three new putative direct ER target genes identified by our stringent criteria for further validation. *NRIP1*, *GREB1*, and *ABCA3 *are all genes found to be ER responsive in at least two cell lines; they have a discernable ERE around the TSS, blocked by ICI and not inhibited by CHX, and their expression can discern ER status in breast cancers. Using ChIP we confirmed that the EREs in all three are directly targeted by ER following estrogen stimulation (Figure 7). Therefore, our process of ranking by consensus (that is, ranking by likelihood of being a direct target of ER by the number of criteria fulfilled) appears to be a reasonable approach to identify actual direct targets of ER.

These target genes suggest potential roles for ER in regulating intracellular signaling pathways that may have an impact on processes in breast and tumor biology. *NRIP1 *was first identified as an ER-binding co-regulator protein and was subsequently found to interact with other nuclear receptors through the nuclear receptor binding motif LXXLL. Kerley and colleagues [56] showed that *NRIP1 *transcript and protein levels were also upregulated by all-*trans *retinoic acid treatment and suggested that *NRIP1 *may facilitate cross-talk between members of the nuclear receptor family. Thus, upregulation of *NRIP1 *by activated ER may not only modulate the estrogen response but also affect the transcriptional activities of other nuclear receptors and the cellular responses to their corresponding ligands. That *NRIP1 *transcript levels were elevated in ER^+ ^compared to ER^- ^breast tumors suggests that the downstream function of other nuclear hormone receptor may be coordinately modulated by elements of the ER transcriptional cascade (see Figure 4).

*ABCA3 *encodes a member of the ABC transporters that utilize ATP hydrolysis to drive the transport of substrates across the cell membrane; although its substrate is not known, ABCA3 appears to be related to other ABC transporters involved in lipid transport. Levels of ABCA3 protein are highest in lung tissue, and ABCA3 appears to localize to lamellar bodies of alveolar epithelial cells that are highly enriched in phosphatidylcholine [57]. These observations provide potential links between ER activation and alterations in phospholipid levels during breast epithelial cell differentiation and transformation. *GREB1*, however, is a gene of unknown function and is unrelated to any other known gene. Its overexpression in ER^+ ^breast tumors and its evolutionary conservation suggest a central role for this gene in ER signaling and breast tumor biology. Of note, 21% (19/89) of the putative target genes identified in this study have no known biological functions (see Table 1).

One strategy used in assessing *cis*-regulatory elements in the genome has been to map conserved segments in non-coding regions upstream of TSSs. Using the three genes above (*NRIP1*, *ABCA3 *and *GREB1*) as rigorously tested direct targets of ER regulation and a well-studied ER direct target, *TFF1/pS2*, we assessed the evolutionary conservation of the validated upstream EREs between human and mouse homologs. Interestingly, we found highly conserved EREs (including flanking regions) only for *NRIP1 *and *GREB1*. *ABCA3 *and *TFF1/pS2 *both have upstream functional EREs in the human genes but not in their mouse orthologs (Figure 7b).

We then extended this search for evolutionary conservation to the remaining 89 putative human ER direct target genes. Surprisingly, we found that in the majority of mouse-human orthologous pairs, the ERE core sequences, flanking regions and position relative to the TSS are not conserved: only 4 out of the testable 72 (6%) orthologous pairs examined showed conservation of ERE sequences between the human and mouse genes. This is remarkable given the 84.7% [58] identity between mouse and human sequences within coding regions. Taken together, our results suggest that the evolution of transcriptional control through *cis*-regulatory mechanisms must have different mutational rates or mechanisms, and may have undergone different selection pressures from those imposed on coding sequences. Moreover, the low level of conservation in the EREs of estrogen-responsive genes between mouse and human suggest two consequences: first, that the core physiologic estrogen effects such as sex differentiation/mammary gland development may be mediated by a small set of highly conserved and similarly regulated ER-responsive genes; and second, that there might be significant differences between downstream estrogen effects between mouse and human.

We suggest the relevance of many of these estrogen-response genes to breast tumor biology by showing significant similarities between estrogen-induced expression profiles in MCF-7 cells and the behavior of these genes in ER^+ ^tumors from a database of six breast cancer microarray studies [36-40]. Not unexpectedly we observed that the number of direct estrogen-responsive genes was small in comparison to the overall number of genes that define the ER^+ ^breast tumors, suggesting that the estrogen-responsive pathways account for only a portion of the receptor-positive molecular signature, an observation also noted by others [22]. Nevertheless, taken together, it appears that the ER^+ ^status of primary breast cancers can be accounted for by concordant effects of an activated ER. Interestingly, a number of these differentially expressed genes (around 30%) were expressed in the opposite direction in the cell lines compared to the tumor consensus. We speculate that this may be due either to consistently different profiles of ER cofactors or to an intense expression signature in tumor-associated stromal cells that is opposite to that of the cancer cells. Nevertheless, those genes that are estrogen-responsive in cell lines and differentially expressed in ER^+ ^tumors represent the most promising candidates for further functional analysis.

In summary, we have presented an integrated strategy for discovering and characterizing ER target genes, response elements and the transcriptional regulatory network downstream of ER activation. With this approach, we uncovered a universal set of genes that describe the most direct effects of ER and operate across multiple *in vitro *and *in vivo *systems. On examination, this core direct target gene list does not predict a unified biological process controlled by ER. Instead, the gene functions would predict a pleiotropic cellular response. By further *in silico *analysis of the promoter regions, we observed minimal conservation in the *cis*-regulatory region of the direct estrogen-response genes between humans and mice. This raises the intriguing possibility that the evolutionary processes governing the configuration of transcriptional regulation will be different from those affecting the functional domains of genes. Moreover, we predict that the estrogen response in the mouse will differ significantly from that in the human, but that a small set of ER direct target genes that are highly conserved in their *cis*-regulatory regions will act as the key effectors of evolutionarily important core ER functions such as sex differentiation.

## Conclusions

Estrogen responses in human breast tumor cells appear to be mediated by a relatively small conserved core set of ER-regulated genes. Examination of the *cis*-regulatory regions of putative target genes within this core set revealed the enrichment of the ERE sequence motif but not other known ER-binding sites. Of all the predicted EREs in human direct target genes, only a handful (6%) appear to be conserved in mouse orthologs, although both conserved and non-conserved predicted EREs were shown to bind ER in human cell lines. Taken together, these findings suggest the potential for species-specific mechanisms and effects in response to hormone exposure.

## Materials and methods

### Cell culture, treatments and RNA extraction

T-47D and MCF-7 cells were maintained in DMEM/F12 (1:1) medium (Invitrogen) supplemented with 10% fetal calf serum (FCS) (Hyclone) at 37°C and buffered with 5% CO_2_. For estrogen treatments, cells were washed with PBS and pre-cultured in phenol-red-free DMEM/F12 medium supplemented with 0.5% charcoal-filtered FCS (Hyclone) for 24 h. For time-course experiments, T-47D cells were treated with 1 nM 17β-estradiol (E2; Sigma-Aldrich) or 1 nM E2 + 10 nM ICI 182, 780 (Tocris Cookson) for the amount of time specified. To determine the primary response, cells were treated with 5 μg/ml cycloheximide (CHX; Sigma-Aldrich) for 30 min before the start of estrogen treatment. Control treatments with ICI (2, 8, 12 and 24 h) and CHX (2 and 8 h) alone were also carried out for the times specified and at the same concentrations as above. To extract RNA, cells were washed with PBS, lysed in Trizol (Invitrogen) and samples were harvested by additional phenol-chloroform extraction steps as prescribed by the manufacturer.

### Microarray analysis of gene-expression profiles

Microarrays were generated by spotting the Compugen 19 K human oligo library, made by Sigma-Genosys, on poly-L-lysine-coated glass slides. Twenty-five micrograms of each sample total RNA and human universal reference RNA (Stratagene) were labeled with Cy5-conjugated dUTP and Cy3-conjugated dUTP (PerkinElmer), respectively, and hybridized to the arrays using protocols established by the Patrick O. Brown Laboratory [59]. Array images and data were obtained and processed using the GenePix4000B scanner and GenePix Pro software (Axon Instruments). Differentially expressed genes were determined using pairwise *t*-test between matching treated samples and mock-treated controls at each time point and fold-difference cutoff at multiple time points as described and clustered and visualized using the Eisen Cluster and TreeView programs [30]. Gene ontology of putative target genes was derived from annotations made by Compugen.

### Meta-analysis of breast cancer and cell line microarray data

A database containing published and unpublished breast cancer expression data was queried for genes whose expression profiles differentiated ER^+ ^and ER^- ^tumors. Each individual dataset was analyzed independently for differentially expressed genes by calculating the false discovery rate for each gene [60] and setting the *p*-value filter at less than or equal to 0.01. ER-status-associated genes were then cross-referenced with the *in vitro *estrogen-responsive genes via the UniGene cluster ID (build 166). The log-transformed average mean-centered expression values for each statistically significant study were used for visualization. Raw *in vitro *MCF-7 estrogen response data [31] were downloaded from the Stanford Microarray Database [32]. The data were compared directly with the T-47D results or selected for ER regulation by the following selection criteria: first, at least a 1.15-fold change in the same direction in two out of three time points and no conflicting (opposite direction) data in any of the time points; and second, changes in the opposite direction when co-treated with tamoxifen (Tam) for 48 h in one out of the two treatment conditions and no conflicting data in the two treatments. The 1.15-fold cutoff, which differs from the 1.2-fold change for the T-47D data, was selected to capture known E2-responsive genes in this dataset.

### Promoter sequence extraction and detection of ER-binding sites

The LocusLink and RefSeq [61] databases at the National Center for Biotechnology Information (NCBI) were used to identify human and mouse genes and pinpoint their loci within the genome. These annotations were chosen for their comprehensiveness, in terms of number of annotated genes, and their consistency with the current state of NCBI contig databases. Using the TSS, defined as the most 5' nucleotide in the reference transcript, and the 3' terminus of the transcript as reference points, we extracted 3 kb upstream and 500 bases downstream of the start sites for binding-site analyses. NCBI human genome sequence build 33 and mouse genome sequence build 30 were used for transcript alignment and genomic sequence extraction. TSS locations annotated in LocusLink and RefSeq may only approximate true start sites because of incomplete information at the 5' ends of some reference sequences, but we believe that the relatively large (3.5 kb) regions used for our analysis allow for fluctuations in TSS position. Human-mouse ortholog determinations were based on annotations made in the Mouse Genome Database [43]. The four binding-site position weight matrix (PWM) models used were either derived in an earlier study [41] or downloaded from the TRANSFAC (version 6.0) database of transcription factor binding sites [42]. Detection parameters were set on the basis of optimized settings for the Dragon ERE Finder [41] at 83% sensitivity in detecting training data and corresponding settings were made for the other PWMs to have similar sensitivities. Statistical significance of binding-site enrichment between putative target genes and non-responsive genes was determined by Monte Carlo simulations between predictions in defined gene sets and randomly generated genes sets. A set of Monte Carlo simulations was performed to assess the significance of the apparent enrichment of putative EREs between the set of estrogen direct target genes and the non-responsive genes. In each simulation, we randomly generated two sets of genes (equivalent in sizes to the set of direct target and non-responsive genes), plotted the curves accordingly, and calculated the difference between the areas under the two curves. The simulations were performed 100,000,000 times and the fraction of times in the simulations that the random area-difference was at least as large as the observed area difference was reported as the empirical *p*-value. Most significant direct target genes used in the analysis were ranked by the lowest *p*-values from analysis of E2-treated and control samples, E2 and E2+ICI samples, and E2 and E2+CHX samples. Non-responsive genes were ranked by highest *p*-values from the same analysis.

### Chromatin immunoprecipitation assays

MCF7 cells were estrogen deprived for 24 h and treated with 100 nM E2 for 45 min before 1% formaldehyde treatment to cross-link the transcription machinery and the chromatin. Immunoprecipitations were carried out overnight with ERα (HC-20) or GST antibodies (Santa-Cruz Biotechnology) and protein A-sepharose beads (Zymed). Washing and extraction protocols were modified from methods described previously [62] and PCR reactions were carried out in a LightCycler (Roche Diagnostics) real-time system. Forty cycles of PCR were carried out on precipitated DNA and control input DNA using the following primer sets: *TFF1/pS2 *ERE: forward CCATGTTGGCCAGGCTAGTC; reverse ACAACAGTGGCTCACGGGGT. *NRIP1 *ERE: forward, TGCTCCTGGGTCCTACGTCT; reverse TCCCCTTCACCCCACAACAC. *GREB1 *ERE: forward AGCAGTGAAAAAAAGTGTGGCAACTGGG; reverse CGACCCACAGAAATGAAAAGGCAGCAAACT. *ABCA3 *ERE: forward, CACCTTCCATCTGTCCAAAG; reverse, CAACCCTGAGGTTTGGGAAC. Actin exon 3 control: forward, AGACCTTCAACACCCCAGCC; reverse, GTCACGCACGATTTCCCGCT. Amplification products were also assayed for specificity by melting-curves analysis at the end of each run. Relative quantifications were carried out by building standard curves for each primer set and using genomic DNA, similar to the input, as the template. Enrichment of ER binding was determined by comparing the relative quantities of anti-ER and control anti-GST products.

## Additional data files

The following additional data files are available with the online version of this article: the processed raw data for the time course microarray study (Additional data file 1) and replicate 1 (Additional data file 2) and replicate 2 (Additional data file 3) of the control microarray study with the ICI and CHX treatments alone, the complete list of all 387 estrogen-responsive genes described in the article with UniGene cluster numbers from build 166 (Additional data file 4), expression profiles of ICI and CHX responsive genes identified in the control experiments (Additional data file 5) and the corresponding figure legend (Additional data file 6).

## Supplementary Material

Additional data file 1 The processed raw data for the time course microarray study Click here for additional data file

Additional data file 2 Replicate 1 of the control microarray study with the ICI and CHX treatments alone Click here for additional data file

Additional data file 3 Replicate 2 of the control microarray study with the ICI and CHX treatments alone Click here for additional data file

Additional data file 4 The complete list of all 387 estrogen-responsive genes described in the article with UniGene cluster numbers from build 166 Click here for additional data file

Additional data file 5 Expression profiles of ICI and CHX responsive genes identified in the control experiments Click here for additional data file

Additional data file 6 The corresponding figure legend to expression profiles of ICI and CHX responsive genes identified in the control experiments Click here for additional data file

## Figures and Tables

**Table 1:** List of putative ER target genes

Accession number	Symbol	Gene name	Number of time points*	Gene ontology
**(a) Putative ER target genes that are upregulated (59 out of 89)**				
AL049265		mRNA; cDNA DKFZp564F053	14	Biological_process unknown [0000004]
NM_003246	*THBS1*	thrombospondin 1	13	Cell adhesion [0007155]
NM_016339	*Link-GEFII*	Link guanine nucleotide exchange factor II	13	Neurogenesis [0007399]
U79299	*OLFM1*	Olfactomedin 1	13	Signal transduction [0007165]
AK026062	*DNAJC1*	DnaJ (Hsp40) homolog, subfamily C, member 1	12	Protein folding [0006457]
M62403	***IGFBP4***^†^	Insulin-like growth factor binding protein 4	12	Signal transduction [0007165]
NM_001089	*ABCA3*^†^	ATP-binding cassette, sub-family A (ABC1), member 3	12	ATP-binding cassette (ABC) transporter [0004009]
NM_003714	*STC2*	Stanniocalcin 2	12	Cell-cell signaling [0007267]
AF075060		Hypothetical protein DKFZp761P0423	11	Biological_process unknown [0000004]
AF271070	*SLC38A1*	Solute carrier family 38, member 1	11	Amino acid transport [0006865]
AK024639		Hypothetical protein FLJ20986	11	Cation transport [0006812]
AL080199		mRNA; cDNA DKFZp434E082	11	Biological_process unknown [0000004]
NM_014365	*H*11	Protein kinase H11	11	Translational regulation, initiation [0006446]
AF245389	***GREB1***^†‡^	GREB1 protein	10	High-affinity zinc ion transport [0006830]
NM_002894	***RBBP8***^†^	Retinoblastoma binding protein 8	10	DNA repair [0006281]
NM_005067	*SIAH2*	Seven in absentia homolog 2 (*Drosophila*)	10	Ubiquitin-dependent protein degradation [0006511]
U16752	***CXCL12***^†^	Chemokine (C-X-C motif) ligand 12 (stromal cell-derived factor 1)	10	Immune response [0006955]
AF086500	*FZD8*	Frizzled homolog 8 (*Drosophila*)	9	Biological_process unknown [0000004]
AF176012	*JDP1*	J domain containing protein 1	9	Physiological processes [0007582]
AF182416	*NIF3L1*	NIF3 NGG1 interacting factor 3-like 1 (S. pombe)	9	DNA methylation [0006306]
AK023772		Hypothetical protein FLJ13710	9	Developmental processes [0007275]
AK024361		Hypothetical protein FLJ14299	9	Transcription regulation [0006355]
AK025812		cDNA: FLJ22159 fis, clone HRC00251, mRNA sequence	9	Biological_process unknown [0000004]
NM_001037	*SCN1B*	Sodium channel, voltage-gated, type I, beta polypeptide	9	Sodium transport [0006814]
NM_003287	*TPD52L1*	Tumor protein D52-like 1	9	Signal transducer [0004871]
NM_003646	*DGKZ*	Diacylglycerol kinase, zeta 104 kDa	9	Signal transduction [0007165]
NM_014333	*IGSF4*	Immunoglobulin superfamily, member 4	9	Virulence [0009406]
NM_016300	*ARPP-21*	Cyclic AMP-regulated phosphoprotein, 21 kD	9	Biological_process unknown [0000004]
AF200341	*HPYR1*	*Helicobacter pylori *responsive 1	8	Biological_process unknown [0000004]
AK023199		cDNA FLJ13137 fis, clone NT2RP3003150, mRNA sequence	8	Biological_process unknown [0000004]
AK025571		Hypothetical protein FLJ21918	8	RNA processing [0006396]
D00265	*CYCS*	Cytochrome c, somatic	8	Electron transport [0006118]
NM_000926	***PGR***^†^	Progesterone receptor	8	Signal transduction [0007165]
NM_001634	*AMD1*	S-adenosylmethionine decarboxylase 1	8	Polyamine biosynthesis [0006596]
NM_002184	*IL6ST*^‡^	Interleukin 6 signal transducer (gp130, oncostatin M receptor)	8	Signal transduction [0007165]
NM_003489	***NRIP1***^†^	Nuclear receptor interacting protein 1	8	Transcription regulation [0006355]
NM_004878	*PTGES*	Prostaglandin E synthase	8	Prostaglandin metabolism [0006693]
NM_012111	*C14orf3*	Chromosome 14 open reading frame 3	8	Protein folding [0006457]
NM_015878	*OAZIN*	Ornithine decarboxylase antizyme inhibitor	8	Polyamine biosynthesis [0006596]
AK023680	*PPP1R15B*	Protein phosphatase 1, regulatory (inhibitor) subunit 15B	7	Biological_process unknown [0000004]
NM_001909	***CTSD***^†^	Cathepsin D (lysosomal aspartyl protease)	7	Proteolysis and peptidolysis [0006508]
NM_003774	*GALNT4*	UDP-N-acetyl-alpha-D-galactosamine:polypeptide N-acetylgalactosaminyltransferase 4	7	Biological_process unknown [0000004]
NM_014810	*CAP350*	Centrosome-associated protein 350	7	Non-selective vesicle transport [0006899]
NM_016391	*HSPC111*	Hypothetical protein HSPC111	7	Leading strand elongation [0006272]
AK021773		cDNA FLJ11711 fis, clone HEMBA1005152, mRNA sequence	6	Biological_process unknown [0000004]
AK025766	*BRI3BP*	BRI3 binding protein	6	Biological_process unknown [0000004]
L23401		Human repeat region mRNA	6	Biological_process unknown [0000004]
NM_000427	*LOR*	Loricrin	6	Cell shape and cell size control [0007148]
NM_001932	*MPP3*	Membrane protein, palmitoylated 3 (MAGUK p55 subfamily member 3)	6	Signal transduction [0007165]
NM_002227	*JAK1*	Janus kinase 1 (a protein tyrosine kinase)	6	Protein phosphorylation [0006468]
NM_006392	*NOL5A*	Nucleolar protein 5A (56 kDa with KKE/D repeat)	6	Transcription [0006350]
NM_006796	*AFG3L2*	AFG3 ATPase family gene 3-like 2 (yeast)	6	Biological_process unknown [0000004]
NM_013324	*CISH*	Cytokine inducible SH2-containing protein	6	JAK-STAT cascade [0007259]
NM_016233	*PADI3*	Peptidyl arginine deiminase, type III	6	Protein modification [0006464]
NM_020120	*UGCGL1*	UDP-glucose ceramide glucosyltransferase-like 1	6	Protein modification [0006464]
AB037842	*KIAA1421*^‡^	KIAA1421 protein	5	RNA dependent DNA replication [0006278]
NM_001116	*ADCY9*	Adenylate cyclase 9	5	Signal transduction [0007165]
NM_014121		PRO0233 protein	5	Double-strand break repair [0006303]
NM_018053		Hypothetical protein FLJ10307	5	Cell death [0008219]
				
**(b) Putative ER target genes that are downregulated (30 out of 89)**				
NM_012342	***NMA***^†^	Putative transmembrane protein	13	Melanin biosynthesis from tyrosine [0006583]
AF039944	*NDRG1*	N-myc downstream regulated gene 1	11	Biological_process unknown [0000004]
AL049471		mRNA; cDNA DKFZp586N012	11	Biological_process unknown [0000004]
AK024964	*NFIA*	Nuclear factor I/A	9	DNA replication [0006260]
M16006	*SERPINE1*	Serine (or cysteine) proteinase inhibitor, clade E, member 1	9	Acute-phase response [0006953]
NM_004438	*EPHA4*	EphA4	9	Signal transduction [0007165]
NM_006449	*CDC42EP3*	CDC42 effector protein (Rho GTPase binding) 3	9	Signal transduction [0007165]
AK026298	*NMES1*	Normal mucosa of esophagus specific 1	8	Biological_process unknown [0000004]
D16875		Human HepG2 3' region cDNA, clone hmd1f06, mRNA sequence	8	Biological_process unknown [0000004]
NM_002237	*KCNG1*	Potassium voltage-gated channel, subfamily G, member 1	8	Potassium transport [0006813]
NM_003032	*SIAT1*	Sialyltransferase 1 (beta-galactoside alpha-2,6-sialytransferase)	8	Protein modification [0006464]
NM_006605	*RFPL2*	Ret finger protein-like 2	8	Protein binding [0005515]
AK025922		Hypothetical protein FLJ22269	7	Developmental processes [0007275]
NM_000504	*F*10	Coagulation factor X	7	Proteolysis and peptidolysis [0006508]
NM_001139	*ALOX12B*	Arachidonate 12-lipoxygenase, 12R type	7	Epidermal differentiation [0008544]
NM_004354	*CCNG2*	Cyclin G2	7	Cell cycle checkpoint [0000075]
NM_006137	*CD7*	CD7 antigen (p41)	7	Humoral defense mechanism [0006959]
NM_007273	*REA*^‡^	Repressor of estrogen receptor activity	7	Negative control of cell proliferation [0008285]
NM_014583	*LMCD1*	LIM and cysteine-rich domains 1	7	Transcription factor [0003700]
NM_017572	*MKNK2*	MAP kinase-interacting serine/threonine kinase 2	7	Protein phosphorylation [0006468]
D49356		Human mRNA (S100C-related gene)	6	Cell cycle [0007049]
NM_001878	*CRABP2*	Cellular retinoic acid binding protein 2	6	Signal transduction [0007165]
NM_004388	*CTBS*	Chitobiase, di-N-acetyl-	6	Carbohydrate metabolism [0005975]
NM_006622	*SNK*	serum-inducible kinase	6	Protein phosphorylation [0006468]
AK022072		cDNA FLJ12010 fis, clone HEMBB1001635, mRNA sequence	5	Biological_process unknown [0000004]
AL137529		Hypothetical protein FLJ23751	5	Lipid metabolism [0006629]
NM_000430	*PAFAH1B1*	Platelet-activating factor acetylhydrolase, isoform Ib, alpha subunit 45 kDa	5	Signal transduction [0007165]
NM_013332	*HIG2*	Hypoxia-inducible protein 2	5	Biological_process unknown [0000004]
NM_014770	*CENTG1*	Centaurin, gamma 1	5	Cell growth and/or maintenance [0008151]
NM_001719	***BMP7***^†^	Bone morphogenetic protein 7 (osteogenic protein 1)	4	Cell growth and/or maintenance [0008151]

*Number of time points that met the 1.2-fold change in the same direction selection criteria. ^†^Genes in bold have previously been shown to be direct targets in other ER^+ ^breast tumor cell lines (ZR75-1 and/or MCF-7). ^‡^CHX treatments alone had agonistic effects on these genes; therefore their CHX sensitivity in the presence of E2 is unclear.

**Table 2:** Comparative analysis of predicted EREs in 22 human and mouse orthologous gene pairs

Gene symbol	Human		Mouse		% Identity*		Relative position* (Δ*d*)
				
	RefSeq ID	Number of predictions	RefSeqID	Number of predictions	Core ERE	Flanking region	
*ALOX12B*	NM_001139	3	NM_009659	4	100	87	40
*NRIP1*	NM_003489	2	NM_173440	4	100	72	70
*GREB1*	NM_014668	2	NM_015764	2	100	72	1,720
*ACP33*	NM_016630	3	NM_138584	4	100	66	46
							
*F10*	NM_000504	2	NM_007972	6	90	49	2,003
*SERPINE1*	NM_000602	4	NM_008871	3	90	23	41
*CTSD*	NM_001909	3	NM_009983	1	90	26	1,310
*OAZIN*	NM_015878	1	NM_018745	1	90	19	2,264
*PADI3*	NM_016233	4	NM_011060	2	90	21	4,153
Unknown	NM_017770	2	NM_019423	1	90	28	3,828
*JDP1*	NM_021800	1	NM_013888	1	90	30	1,089
*NIF3L1*	NM_021824	1	NM_022988	2	90	28	955
							
*SCN1B*	NM_001037	2	NM_011322	1	80	36	2,716
*STC2*	NM_003714	1	NM_011491	2	80	26	2,638
							
*LOR*	NM_000427	2	NM_008508	2	70	32	4,253
*ADCY9*	NM_001116	4	NM_009624	1	70	28	3,042
*AHCYL1*	NM_006621	1	NM_145542	2	70	32	2,190
*CISH*	NM_013324	1	NM_009895	3	70	19	1,624
*HSPC111*	NM_016391	1	NM_178605	1	70	19	1,302
*FLJ22269*	NM_032219	2	NM_172883	1	70	47	3,391
							
*DNAJC1*	NM_022365	1	NM_007869	1	60	30	1,672
*CDC42EP3*	NM_006449	1	NM_026514	1	50	25	451

*The % identity and relative positions of EREs refer to the predicted pairs with the highest conservation between the two organisms.
