# Supplementary figures and images for: Discovery of estrogen receptor α target genes and response elements in breast tumor cells
Source: Genome Biol. 2004 Aug 12;5(9):R66. doi: 10.1186/gb-2004-5-9-r66 (PMC522873; doi:10.1186/gb-2004-5-9-r66)

A

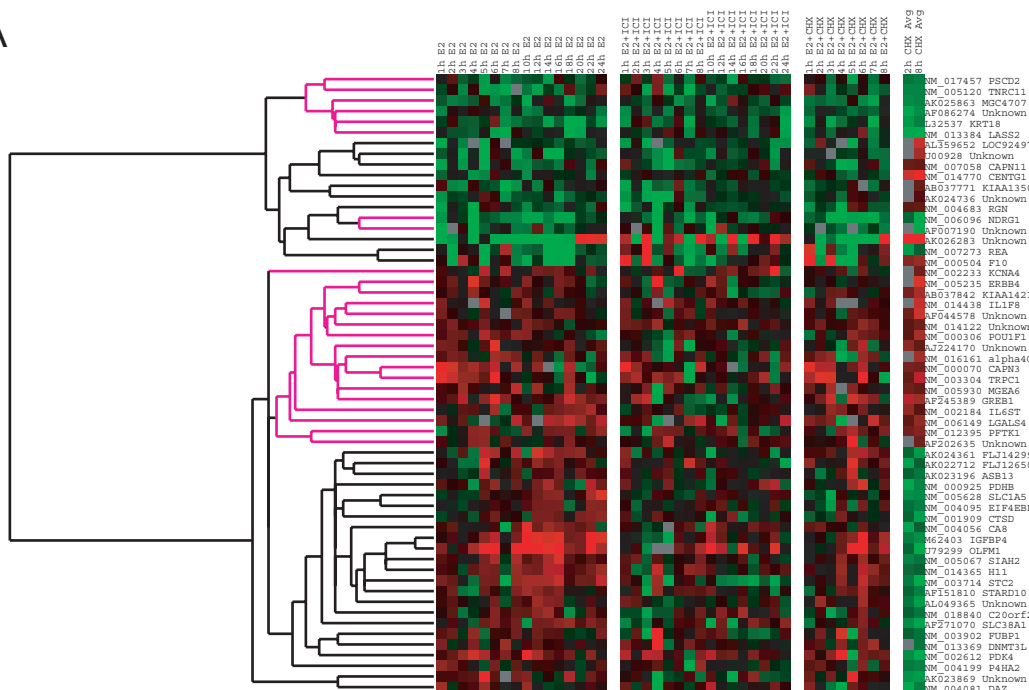

B

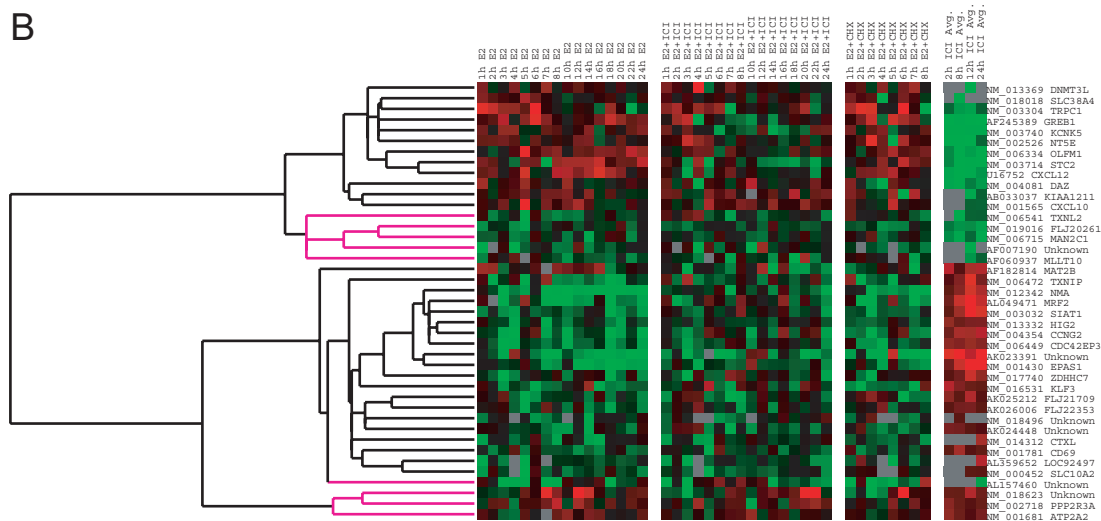

Supplement: Additional data file 5 — Expression profiles of ICI and CHX responsive genes identified in the control experiments [file gb-2004-5-9-r66-s5.pdf]
