# Supplementary material for: Discovery of estrogen receptor α target genes and response elements in breast tumor cells
Source: Genome Biol. 2004 Aug 12;5(9):R66. doi: 10.1186/gb-2004-5-9-r66 (PMC522873; doi:10.1186/gb-2004-5-9-r66)
Supplement: Additional data file 6 — The corresponding figure legend to expression profiles of ICI and CHX responsive genes identified in the control experiments [file gb-2004-5-9-r66-s6.doc]

Supplementary Figure 1. CHX (A) and ICI (B) treatments alone induces responses in E2 responsive genes. Gene trees of genes with similar responses to E2 and CHX or ICI are highlighted in magenta. 26 of 386 E2 responsive genes are similarly responsive to CHX treatment alone, including 4 putative direct target genes. ICI treatment alone elicited the same response as E2 in 9 responsive genes. To capture the overlap between E2 response and CHX and ICI alone effects, responsive genes, were defined liberally by a 1.15 fold change in at least one time point and no contradictory responses in other time points.
